# Sequence-ensemble-function relationships for disordered proteins in live cells

**DOI:** 10.21203/rs.3.rs-3501110/v1

**Published:** 2023-11-10

**Authors:** Alex Holehouse, Ryan Emenecker, Karina Guadalupe, Nora Shamoon, Shahar Sukenik

**Affiliations:** Washington University in St. Louis; UC Merced; UC Merced; UC Merced

## Abstract

Intrinsically disordered protein regions (IDRs) are ubiquitous across all kingdoms of life and play a variety of essential cellular roles. IDRs exist in a collection of structurally distinct conformers known as an ensemble. IDR amino acid sequence determines its ensemble, which in turn can play an important role in dictating molecular function. Yet a clear link connecting IDR sequence, its ensemble properties, and its molecular function in living cells has not been systematically established. Here, we set out to test this sequence-ensemble-function paradigm using a novel computational method (GOOSE) that enables the rational design of libraries of IDRs by systematically varying specific sequence properties. Using ensemble FRET, we measured the ensemble dimensions of a library of rationally designed IDRs in human-derived cell lines, revealing how IDR sequence influences ensemble dimensions *in situ*. Furthermore, we show that the interplay between sequence and ensemble can tune an IDR’s ability to sense changes in cell volume - a *de novo*molecular function for these synthetic sequences. Our results establish biophysical rules for intracellular sequence-ensemble relationships, enable a new route for understanding how IDR sequences map to function in live cells, and set the ground for the design of synthetic IDRs with *de novo* function.

## Introduction

**Figure 1:**
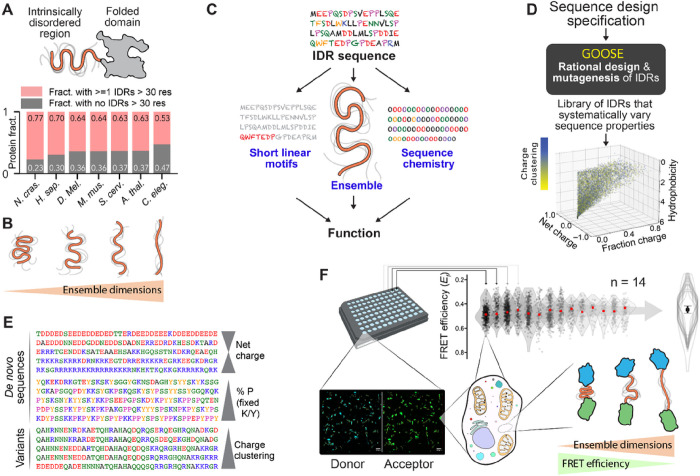
IDR ensemble properties can be important determinants of function. **(A)** IDRs are protein regions that lack a stable 3D structure and are ubiquitous across eukaryotic proteomes. **(B)** While IDRs lack a fixed 3D structure, their conformational behavior can be quantified in terms of ensemble dimensions. **(C)** Emerging work suggests that IDR function depends on an interplay between three key features: sequence chemistry, ensemble properties, and the presence of short linear binding motifs (SLiMs). **(D)** GOOSE is a computational tool for the rational design or mutagenesis of IDRs. In the scatter plot, each point represents a sequence designed by GOOSE to match the constraints defined by the axes and in the color bar. **(E)** GOOSE enables specific sequence parameters to be titrated while others are held fixed. **(F)** An in-cell fluorescence reporter assay combined with a library of rationally designed sequences enables us to determine how IDR sequence properties dictate ensemble properties in the cellular environment (bottom right). Experiments are performed using a 96-well imaging plate (top left), and each well with 60 or more cells is analyzed to calculate FRET efficiency (E_f_) based on donor and acceptor fluorescence (bottom left). Across multiple independent measurements (14 in this case), average E_F_ values are calculated to provide a statistically rigorous and highly reproducible assessment of sequence-specific FRET efficiency (top right). Only the summary violin plot overlays shown here are reported in all other figures, with the average median shown as a circle and the SD of medians as error bars.

Intrinsically disordered proteins and protein regions (IDRs) are ubiquitous across eukaryotic proteomes (Fig. 1A)^1^. Unlike folded domains, IDRs do not adopt a stable 3D structure but exist instead in a collection of interconverting conformations referred to as an ensemble^1–3^. Despite lacking a fixed structure, the chemistry encoded by an IDR’s amino acid sidechains can determine local and long-range conformational biases in the underlying ensemble, all of which work together to determine overall ensemble dimensions (Fig. 1B)^1,3^. Moreover, these conformational biases can influence or even determine IDR function. The average conformational properties of IDR ensembles have been shown to tune binding to partner proteins^4^, refolding of tethered folded domains^5^, enzymatic reaction rates^6^, and the ability to form higher-order oligomers and condensates^7^. Thus, IDRs are disordered but not unstructured, and an emerging paradigm suggests that IDR function depends on a combination of sequence features, ensemble properties, and linear motifs (Fig. 1C).

Various sequence features have emerged as important determinants of IDR conformational behavior^1,3^. The presence of charged residues and how they are arranged^8–12^, the presence of aromatic residues^7^, and the overall hydrophobicity have all been shown to tune intramolecular interactions within the ensemble, alter IDR transient secondary structure, and affect global ensemble dimensions^13,14^. These effects suggest there exists a complex but interpretable mapping between amino acid sequence and the underlying conformational ensemble.

To date, the mapping between IDR sequence and conformational ensemble has primarily been elucidated *in vitro* and *in silico*, yet whether those same rules extend to the cellular environment remains unknown. This is of particular relevance given that IDRs are inherently sensitive to their solution environment^15,16^. Furthermore, the investigation of sequence-ensemble-function relationships in living cells has had limited direct exploration. We therefore sought to examine how IDR sequence-ensemble-function relationships manifest in living cells.

To enable the systematic investigation of sequence-ensemble-function relationships, we developed and deployed a novel approach (GOOSE) for the rational design of IDRs with specific sequence properties (Fig. 1D, E). We used GOOSE to create a library of 32 sequences that explore the sequence-to-ensemble relationship of IDRs through pairwise sequence comparisons.

Prior *in vitro* and *in silico* work identified rules that govern how IDR sequence dictates ensemble dimensions, the average volume occupied by the ensemble (often quantified in terms of the ensemble-average radius of gyration, end-to-end distance, or hydrodynamic radius) (Fig. 1B)^3,8–11,17–19^. To address this question in live cells, we used ensemble Förster Resonance Energy Transfer (FRET) to measure the ensemble dimensions of our designed library. We found that – despite several exceptions – many of the rules elucidated *in vitro* hold under physiological conditions (Fig. 1F). We also show that ensemble dimensions, rather than amino acid composition or sequence, are the strongest determinant of whether an IDR will be responsive to changes in cellular volume. In addition to offering direct support for understanding sequence-ensemble-function relationships in living cells, our work provides an accessible, easy-to-use tool for the rational design of synthetic disordered proteins, sequence variants, and large-scale libraries. This opens the door to high-throughput systematic exploration of sequence-ensemble-function relationships in IDRs.

## Results

### GOOSE enables the rational design of disordered regions

A major conceptual challenge in exploring IDR ensemble-to-function relationships is the difficulty of exploring IDR sequence space. For folded proteins, single-point mutations directly test sequence-structure relationships. In IDRs, single-point mutations are generally expected to have a negligible impact on conformational behavior (**Fig. S1**), and the reduced sequence conservation across homologous proteins suggests that single-point mutations often have a limited impact on function^1,20–22^. In contrast, decoding sequence-ensemble relationships has been effective through mutations that enable titration of sequence properties (e.g., tuning the net charge per residue), but this has been difficult to perform systematically without perturbing multiple sequence features at once^17,20,22–25^. Moreover, the rational design of IDRs with specified ensembles has historically been impossible until very recently^26^. To enable the holistic investigation of sequence-ensemble-function relationships in IDRs, we developed GOOSE. GOOSE is a Python-based software package for the rational design of synthetic sequences or sequence variants based on user-defined parameters (see **Methods** and **Table S2**). Parameters here include specific sequence features but also predicted ensemble dimensions (e.g., the radius of gyration or end-to-end distance).

As a proof of concept, we used GOOSE to design a library of 32 IDRs that allowed us to systematically test the impact of charged residues and hydrophobicity on ensemble dimensions (**Table S1, Fig. S2**). All IDRs were 60 residues in length and were designed to enable the comparison between pairs of sequences that vary specific sequence properties while holding others fixed (**Table S3**). We used a recently developed genetically encoded FRET backbone to profile the dimension of these sequences in live cells^15,27^. Briefly, by placing each IDR between two fluorescent proteins that form a FRET donor and acceptor pair, FRET efficiency becomes a proxy for the IDR’s ensemble-averaged end-to-end distance (i.e. ensemble dimensions); if the ensemble is compact, transfer efficiency is high, and if the ensemble is extended transfer efficiency is low (Fig. 1F). In this way, we assessed how varying different sequence properties altered ensemble-average conformational behavior for IDRs in cells (**Fig. S3**, see also discussion on limitations of the method in Supplementary Information).

### Charged residues can compact or expand ensemble dimensions

**Figure 2:**
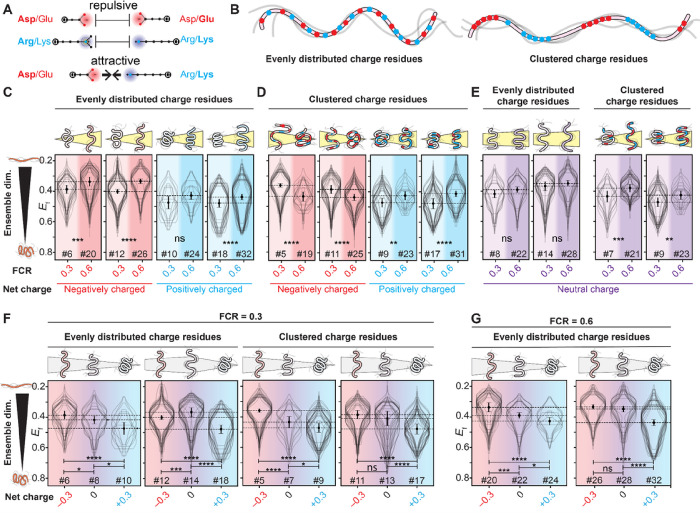
Charged residues influence IDR dimensions in live cells. **(A)** Charged residues of the same polarity are repulsive, while charged residues of opposite polarity are attractive**. (B)** We focus on sequences in which charged residues are either evenly distributed across the sequence (left) or clustered together with like charges (right). **(C)** FRET efficiency E_f_ for IDRs with evenly distributed charged residues and a net charge of −0.3 or +0.3, increasing the fraction of charged residues (FCR) in the sequence leads to ensemble expansion, in line with prior *in vitro* work. **(D)** E_f_ for IDRs with clustered charged residues and a net charge of −0.3 or +0.3, increasing the percentage of charged residues leads to compaction for negatively charged sequences and expansion for positively charged sequences. For all four pairs, the FCR=0.3 sequences contain only one type of charged residue, while the FCR=0.6 sequences contain both. **(E)** For IDRs with a net neutral charge, increasing the fraction of charged residues when charged residues are evenly spaced or clustered leads to, at most, a small increase in IDR dimensions. **(F)** For IDRs with an FRC of 0.3, systematically titrating the net charge from −0.3 to 0 to +0.3 leads, in most cases, to a gradual compaction. The two sets where there is a small expansion (12 to 14) or non-significant compaction (11 to 13) involve the acidic sequence possessing several aromatic residues, likely making it more compact Page 16/18 than it might otherwise be. **(G)** For IDRs with 60% charged residues, systematically titrating the net charge from −0.3 to 0 to +0.3 leads, in most cases, to a gradual compaction.

We first designed sequences that test the effects of charged residues on ensemble dimensions. The importance of charged residues is driven by a combination of the long-range nature of electrostatic interactions and their favorable interaction with the solvent (Fig. 2A)^8–10,28^. Since the intracellular environment drastically differs from dilute aqueous buffers in terms of their ionic composition, we sought to determine whether rules of thumb established in *in vitro* and *in silico* studies are recapitulated in cells.

We first asked whether increasing the fraction of charged residues (FCR) would alter IDR dimensions. Based on *in vitro* studies, we expect that increasing the FCR should lead to ensemble expansion^9,10,29^. To test this hypothesis, we designed sequence pairs where the FCR varied, but other features (*e.g.,* net charge, hydrophobicity, charge patterning, Fig. 2B) were held fixed. In line with prior work, both polyanionic (negatively charged) and polycationic (positively charged) IDRs with evenly distributed charged residues become more expanded as the FCR increased (Fig. 2C)^10,29^.

Next, we wondered how charge patterning might alter this behavior^11^. *In vitro* and *in silico* work has established that clustering oppositely charged residues in an IDR can lead to intra-molecular interactions and ensemble compaction (**Fig. S4**)^11^. We tested if that behavior persists in cells using new sequence pairs that had identical sequence features to those in Fig. 2C, except that charged residues of the same sign were clustered together (Fig. 2B). In line with expectations, increasing the FCR for sequences with charge clusters led to ensemble compaction for IDRs with a net negative charge (Fig. 2D). In contrast, for IDRs with a net positive charge and charge clusters, increasing the FCR led to ensemble expansion, an unexpected result that we speculate may be driven by these polycationic IDRs interacting with other cellular components (see *Discussion*). Taken together, our results show that for IDRs with a net charge, both the FCR and the patterning of charged residues can strongly influence IDR dimensions in cells.

Having examined IDRs with a net positive or negative charge (polyelectrolytes), we next considered polyampholytes, sequences with an equal fraction of positive and negative residues, making them net neutral. In agreement with prior *in vitro* and *in silico* work, we saw no statistically significant change in ensemble dimensions upon an increase in the FCR for polyampholytes with evenly distributed charged residues (Fig. 2E) ^12,17^. However, when oppositely charged residues were clustered together, an increase in the FCR led to an expansion of ensemble dimensions, as was seen for sequences with a net positive charge (Fig. 2E). This unexpected result further points to the importance of positively charged clusters, rather than an overall positive charge, in altering ensemble dimensions. This may again be due to interactions of such positive charge clusters with cellular components (see *Discussion*).

Finally, we asked how IDR ensembles with equivalent FCR values but different charge signs (negative, neutral, or positive) behaved. We designed sequence triplets with an FCR of 0.3 in which the net charge per residue varied (from −0.3 to 0.0 to +0.3), with sets having either evenly distributed or clustered charged residues. *In silico* and *in vitro* predict that the ensemble dimensions of IDRs with the same charge but with opposite signs should display similar dimensions^8–10,12,17^. To our surprise, this behavior was not recapitulated in our experiments (Fig. 2F). In two of the four triplets, we saw a monotonic decrease in IDR dimensions as positively charged residues were added. Moreover, in all four cases, the IDR with a +0.3 net charge was the most compact of the three sequences. This trend held true for a second set of sequence triplets with an FCR of 0.6 (Fig. 2G). In summary, these results suggest that IDRs with a net positive charge are more compact than equivalent IDRs with a neutral or negative charge.

Taken together, while these experiments do identify several differences between *in vitro* and in-cell observations, our results largely confirm that the same properties that influence IDR dimensions *in vitro* hold true in cells. Increasing the fraction of charged residues makes polyelectrolytic IDRs (sequences with a net charge) expand, while this effect is more muted for polyampholytic IDRs (sequences without a net charge). Most notably, negative polyelectrolytes are highly expanded, while positive polyelectrolytes are unexpectedly compact.

### Modest increases in hydrophobicity do not lead to IDR compaction in cells

Having established that charged residues are key determinants of IDR dimensions in cells as observed *in vitro*, we next sought to assess how sequence hydrophobicity influences IDR dimensions in cells. The role of hydrophobicity on the global dimensions of unfolded and disordered proteins has received substantial attention^30–33^. In the protein folding literature, understanding the impact of hydrophobicity on the ensemble dimensions of unfolded polypeptides under folding conditions has been hampered historically by technical challenges but, more recently and perhaps more fundamentally, the limitation that aqueous buffer is not bona *fide* “native conditions”^33,34^.

**Figure 3:**
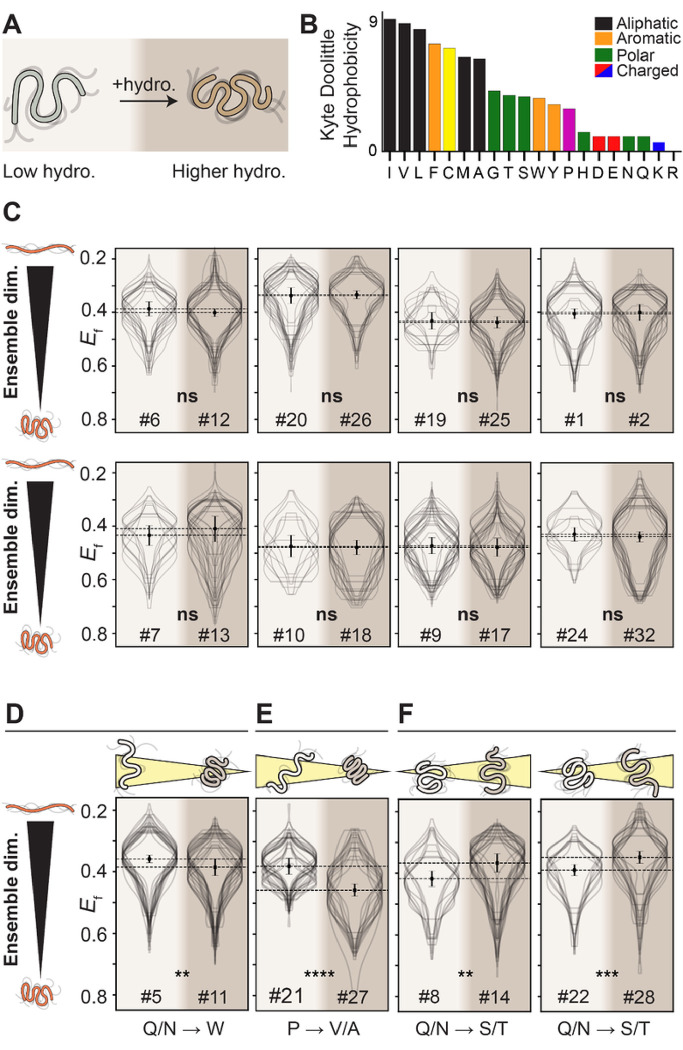
Moderate changes in hydrophobicity have, in general, no impact on IDR dimensions. **(A)** For all hydrophobicity designs, the pair of sequences varies the Kyte-Doolittle hydrophobicity from 1 to 3. **(B)** The Kyte-Doolittle hydrophobicity scale. **(C)** For the majority of sequences tested, despite very different chemical backgrounds, increasing the hydrophobicity from 1 to 3 does not lead to a significant change in IDR dimensions, implying that across the range of hydrophobicity, the cytosol is a good solvent for IDRs. **(D)**One outlier in this insensitivity to changes in hydrophobicity is a small degree of compaction seen, which can be explained by the insertion of tryptophan (W) residues within a charge-depleted region. **(E)** Another outlier inserts aliphatic residues within charge blocks and involves the loss of proline (P), both of which are expected to contribute to increased ensemble compaction. **(F)** The only outliers where we see expansion reflect sequences where glutamine and asparagine (Q/N) are converted to serine and threonine (S/T).

We hypothesized that increasing IDR hydrophobicity would lead to measurable compaction of IDR dimensions in cells (Fig. 3A). To test this hypothesis, we designed twelve pairs of IDRs where hydrophobicity (as defined using the widely-used Kyte-Doolittle hydropathy scale^35^) increases between the two pairs while many other sequence properties remain fixed (Fig. 3B). For the majority (8/12) of the pairs, increasing hydrophobicity had no impact on IDR dimensions, despite a wide range of sequence backgrounds (Fig. 3C). Beyond the implications for the impact of hydrophobicity, this result also indicates that IDRs with different sequences can have indistinguishable ensemble properties, at least as measured in our assay.

For the four pairs where a change in ensemble dimensions was observed, molecular explanations for the differences are readily available. In one pair where increasing hydrophobicity leads to compaction, the specific sequence changes involve inserting three aromatic tryptophan residues into a subregion devoid of any charged residues (Fig. 3D). This result supports prior work showing a linear response between IDR dimensions and aromatic residue content^7^. For another pair, the increase in hydrophobicity accompanies the loss of proline residues along with large bulky aliphatic residues being inserted within two opposite charge blocks (Fig. 3E). Both of these changes are expected to drive ensemble compaction independently, such that the combined effect of both may be cooperative^13,36^. Finally, two of the pairs show an increase in IDR dimensions upon an increase in hydrophobicity, driven by the exchange of glutamine and asparagine residues for serine and threonine. Prior work has shown glutamine and asparagine can drive attractive interactions via the secondary amide group, whereas serine and threonine are not expected to interact as strongly (Fig. 3F)^37^. In summary, while specific and interpretable sequence features may diverge from the overall trend, our results here suggest that increasing IDR hydrophobicity within the bounds explored here does not, in general, lead to ensemble compaction.

### Ensemble dimensions predict IDR function

Given their solvent-exposed nature and the small number of intramolecular bonds that dictate ensemble conformational biases, IDRs are poised to respond to changes in their physicochemical surroundings through a change in their ensemble dimensions^15,16,27,38^. We reasoned that we could use our GOOSE-generated library to test how sequence properties map to their sensing ability.

**Figure 4:**
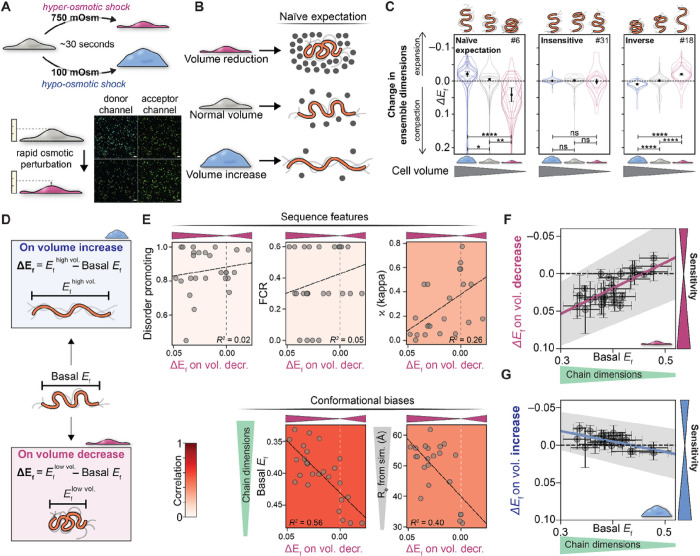
IDR ensemble dimensions explain responsiveness to changes in cell volume. **(A)** Using rapid osmotic perturbation, cellular volume is reduced (top) or expanded (bottom), altering intracellular crowding within Page 18/18 seconds. These timescales ensure changes in FRET efficiency (ΔE_f_) reflect physical changes in the cellular environment, as opposed to the downstream consequences of a response pathway driven by phosphorylation or gene expression. **(B)** If macromolecular crowding were the only determinant of IDR dimensions, we would naively expect a decrease in cell volume to drive ensemble compaction (top) and an increase in cell volume to drive ensemble expansion (bottom). **(C)** Actual data for sequences 5/31/18, demonstrating that while some sequences respond as expected by the naive model (left), others show complete insensitivity (middle) while others show inverse behavior, expanding upon volume reduction (right). These results illustrate the complex range of sequence-dependent responses available to IDRs. **(D)** To quantitatively compare different sequences, we can compare basal FRET efficiency (E_f_ under normal conditions) with the change in FRET efficiency ΔE_f_ upon increase or decrease in cellular volume. **(E)** Sequence properties (fraction of disorder-promoting residues, FCR, and kappa) are weakly correlated with molecular function (see also **Fig. S10**), here defined as the change in FRET efficiency upon cell volume decrease. In correlating ΔE_f_ vs. many sequence parameters, E_f_ basal (and, to a lesser extent, ensemble dimensions from coarse-grained simulations) offer the strongest predictive power of molecular function (**Fig. S10**). **(F)** Plotting ΔE_f_ upon the increase in cellular volume and **(G)** ΔE_f_ upon the decrease in cellular volume reveals that the most sensitive sequences (those with the largest change in E_f_) are strongly correlated with those that start out being more expanded under basal conditions.

We used live-cell ensemble FRET to determine how each of our synthetic IDRs responded to cell volume changes induced by hyper- and hypo-osmotic shock (Fig. 4A). Osmotic perturbations are sufficiently fast (~30 seconds) that changes in FRET efficiencies reflect a consequence of the immediate change in cellular environment, as opposed to a secondary effect driven by signaling or transcriptional changes^39^. Sensing cellular volume changes is required for various regulatory and homeostatic processes, including cell division and growth, yet how this is accomplished at the molecular level remains unclear^40^. We hypothesized that an IDR-based cellular volume sensor would alter its ensemble when the cell experiences volume-induced changes. One mechanism often invoked to explain this is that a decrease in cell volume would increase macromolecular crowding in the cytoplasm, which in turn would drive IDR compaction^41^. Cell volume increase should have the opposite effect (Fig. 4B). With this mechanism in mind, we measured sequence sensitivity as the change in FRET efficiency following cell volume increase or decrease (ΔE_f_) in all 32 constructs in our library (**Fig. S5**).

Our results indicate that not all IDR sequences tested here can act as sensors of the cellular environment. Instead, sequences fell into three behavior types: naive (in line with the macromolecular crowding mechanism described above - cell volume reduction causes ensemble compaction), insensitive (non-responsive to volume changes), and inverse (cell volume reduction causes ensemble expansion) (Fig. 4B, C). Around one-quarter of the sequences with sufficient statistics showed naive behavior, at least half showed insensitive behavior, and a fifth showed inverse behavior (**Fig. S6**).

What about our library sequences determines their response to cell volume change? We proposed three possible explanations: (1) that changes in subcellular localization, whether in basal conditions or as a result of volume change, would alter ensemble dimensions and, therefore, predict sequence behavior; (2) that sequence features, such as NCPR, FCR, and other features that were varied (Figs. 2 and 3) dictate sequence response; or (3) that ensemble dimensions are the key determinant for sequence response. We test each of these hypotheses below.

We first hypothesized that nuclear localization would expose sequences to a different environment with different chemistry and molecular composition, which could explain the differences in response to cell volume changes. Indeed, a subset of our sequences (n=10) show nuclear localization – likely mediated by tracts of positively charged residues that are recognized by the nuclear import machinery^42^ (**Fig. S7**). Despite this, almost all sequences had a similar ratio of in the cytoplasm vs in the nucleus (**Fig. S8**), and the ratio of between protein in the nucleus and cytoplasm showed little correlation with the response of the ensemble to changes in cell volume, (**Fig. S9**). We therefore sought to ask if there were simple sequence features that could explain why different sequences showed different responses to cell volume change.

We hypothesized that specific sequence features (**Fig. S10A**) may govern the response to changes in cell volume. To test this, we correlated the change in FRET upon change in cell volume (ΔE_f_) with a range of sequence features, but no strong correlations were found (Fig. 4E top, **Fig. S10B** blue dashed region). We were surprised to find that even charge properties, which showed a strong effect in sequence pairs, had little correlation with the response to volume change. Thus, our data shows that, in this case, molecular function cannot be predicted directly from the sequence features examined here.

Finally, we hypothesized that the response to volume change may be predictable based on IDR ensemble dimensions under iso-osmotic conditions. To test this, we compared the change in FRET upon change in cell volume (ΔE_f_) to the basal FRET efficiency (E_f_) of the construct (Fig. 4E, Fig. 4F, G). The basal FRET efficiency showed a stronger correlation with ΔE_f_ than any other feature. To summarize this result, sequences that are more expanded prior to a change in cell volume are more sensitive to both volume increase and decrease (Fig. 4F, G, and **Fig. S10B**, black dashed region). This result is mirrored by results from simple coarse-grained simulations, where ensemble dimensions are predictive of an IDR’s responsiveness to cell volume perturbation (Fig. 4E, **Fig. S11**).

Our work reveals that, for this system, any single sequence feature is a poor determinant of function. Instead, the best correlation is entirely dependent on the average physical dimensions of the IDR ensemble. These results offer biophysical insight into the molecular basis for IDR sensitivity with implications for the design of *de novo* disordered sensors^43^. More broadly, they directly point to the potential for an intimate relationship between IDR ensemble properties and molecular function in living cells.

## Discussion & Conclusion

Prior work has proposed a sequence-ensemble-function relationship for IDRs, where the amino acid sequence influences the ensemble properties in an interpretable and predictable way. Our experiments show that sequence-ensemble relationships previously observed *in silico* and *in vitro* generally persist inside the cell. Specifically, charge interactions carry an outsized effect on ensemble dimensions, and for sequences with a net negative charge, the overall charge density correlates well with ensemble dimensions. Moreover, sequences where oppositely charged residues are dispersed throughout the sequence tend to be more expanded than those where charged residues of the same type are clustered together.

An unexpected exception to prior observations is that sequences with a net positive charge are generally more compact than their negatively charged counterparts (Fig. 2). This exception occurs not only at the ensemble level: positively charged sequences also tend to display an inverse response to cellular volume change, expanding when cell volume decreases (Fig. 4). One possible explanation for this could be the presence of secondary structure, but this does not bear out in our analysis (**Fig. S12**). An alternative explanation could be interaction with negatively charged cellular components. Indeed, biomacromolecules (including phospholipids, nucleic acids, and even the human proteome) tend to be negatively charged^44,45^. Recent proteome-wide analysis across various species coupled with extant quantitative mass spectrometry found very few cases of highly abundant IDRs with a strong net positive charge^46^. The few IDRs that did contain a strong positive charge were expected to be constitutively bound to nucleic acids, anchored in phospholipid bilayers, or at least partially neutralized via phosphorylation. Based on these independent observations, we speculate that IDRs with a strong net positive charge unavoidably recruit polyanions, leading to ensemble compaction driven by intermolecular charge neutralization (complex coacervation). This interpretation is at least consistent with the “polycation poisoning” model proposed by *Boeynaems et al*.^46^.

Our results also argue that increasing IDR hydrophobicity – at least over the range examined here (**Fig. S13**) – does not inherently drive ensemble compaction in the cellular environment. This mirrors conclusions reached by independent *in vitro* studies and is consistent with a model in which protein folding and hydrophobic collapse are generally distinct molecular processes^12,31,33,34^. Importantly, our work provides strong evidence that biophysical conclusions regarding protein-solvent interactions drawn *in vitro* or *in silico* can hold true in cells.

In addition to developing an intracellular reference for sequence-ensemble relationships, our work highlights the connection between IDR ensemble and molecular function - in this case, the ability to tune ensemble dimension in response to cellular volume change. While this is largely a synthetic function, recent work has highlighted scenarios in which IDR dimensions can play key functional roles^4,12,47^. Our work shows that – by far – the best correlation between this sensing function and any of the metrics we quantified is observed for ensemble conformational properties - and predicted ensemble dimension (**Fig. S10B**). This finding emphasizes the importance of an IDR’s conformational ensemble for cellular function and prompts further studies to explore ensemble-function relationships. Importantly, our results imply that IDRs can be engineered by tuning the ensemble dimensions of the sequence. In this case, the fact that the sensitivity of the end-to-end distance is a sequence-encoded tunable molecular function offers a valuable opportunity for engineering biosensors and actuators, which we have previously used to design disordered sensors for water stress based on a naturally occurring plant IDR^43^. These sensors can effectively utilize this tunable molecular function to “program” IDRs, enabling them to accurately report on specific environmental cues.

In summary, combining rationally designed sequences with ensemble FRET microscopy in live cells allows us to study IDRs in their “native environment”. We have shown that overall, the sequence-ensemble rules decoded *in silico* and *in vitro* hold up in live cells. Furthermore, our work suggests that when it comes to an IDR’s responsiveness to changes in cell volume, ensemble conformational properties – not amino acid composition or patterning – are the main determinants of sensitivity (**Fig. S10B**).

Beyond the specific sequences examined here, our work illustrates how our computational method GOOSE can be leveraged to systematically investigate sequence-ensemble-function relationships in IDRs. Recent work complementary to ours has employed alternative strategies for the design of IDRs based on matching overall bulk properties or using molecular simulations^26,48^. While these approaches offer unique and exciting opportunities for IDR-based synthetic biology, a feature offered by GOOSE is the ability to systematically vary individual sequence features in an “unbiased” way, either via fully synthetic sequences or controlled variants (for both amino acid properties and ensemble properties, **Fig. S14**). While our work here focused on a relatively small library of sequences, GOOSE enables the design of large libraries that systematically vary sequence parameters in an interpretable way^17^. With this in mind, we see GOOSE as being highly complementary to high-throughput experiments to map sequence-function relationships across a wide range of IDRs.
